# Human Papillomavirus (HPV) seroprevalence, cervical HPV prevalence, genotype distribution and cytological lesions in solid organ transplant recipients and immunocompetent women in Sao Paulo, Brazil

**DOI:** 10.1371/journal.pone.0262724

**Published:** 2022-01-20

**Authors:** Karina Takesaki Miyaji, Vanessa Infante, Camila de Melo Picone, José Eduardo Levi, Ana Carolina Soares de Oliveira, Amanda Nazareth Lara, Maricy Tacla, Joakim Dillner, Hanna Kann, Carina Eklund, Cristina Paula Castanheira, Philippe Mayaud, Ana Marli Christovam Sartori, the Study group on HPV vaccine in immunosuppressed persons

**Affiliations:** 1 Departamento de Molestias Infecciosas e Parasitarias da Faculdade de Medicina da Universidade de Sao Paulo (FMUSP), Sao Paulo, Brazil; 2 Clinica de Molestias Infecciosas e Parasitarias do Hospital das Clinicas da Faculdade de Medicina da Universidade de Sao Paulo (HC-FMUSP), Sao Paulo, Brazil; 3 Centro de Referencia para Imunobiológicos Especiais, HC-FMUSP, Sao Paulo, Brazil; 4 Laboratório de Investigação Medica – Virologia, Instituto de Medicina Tropical (IMT)/HC, FMUSP, Sao Paulo, Brazil; 5 Clínica de Ginecologia, HC-FMUSP, Sao Paulo, Brazil; 6 Department of Laboratory Medicine, Karolinska Institute, Stockholm, Sweden; 7 London School of Tropical Medicine and Hygiene (LSTMH), London, England; Women’s Hospital, School of Medicine, Zhejiang University, Hangzhou, China, CHINA

## Abstract

**Introduction:**

Solid organ transplant (SOT) recipients are at increased risk of Human Papillomavirus (HPV) persistent infection and disease. This study aimed to evaluate HPV seroprevalence, cervical HPV prevalence, genotype distribution, and frequency of HPV-related cervical lesions in SOT recipients in comparison to immunocompetent women.

**Methods:**

Cross-sectional study including SOT and immunocompetent women aged 18 to 45 years who denied previous HPV-related lesions. Cervical samples were screened for HPV-DNA by a polymerase chain reaction (PCR)-based DNA microarray system (PapilloCheck^®^) and squamous intraepithelial lesions (SIL) by liquid-based cytology. A multiplexed pseudovirion-based serology assay (PsV-Luminex) was used to measure HPV serum antibodies.

**Results:**

125 SOT and 132 immunocompetent women were enrolled. Cervical samples were collected from 113 SOT and 127 immunocompetent women who had initiated sexual activity. HPV-DNA prevalence was higher in SOT than in immunocompetent women (29.6% vs. 20.2%, *p* = 0.112), but this difference was not statistically significant. High-risk (HR)-HPV was significantly more frequent in SOT than in immunocompetent women (19.4% vs. 7.9%, *p* = 0.014). Simultaneous infection with ≥2 HR-HPV types was found in 3.1% of SOT and 0.9% of immunocompetent women. HPV seropositivity for at least one HPV type was high in both groups: 63.8% of 105 SOT and 69.7% of 119 immunocompetent women (*p* = 0.524). Low-grade (LSIL) and high-grade SIL (HSIL) were significantly more frequent in SOT (9.7% and 5.3%, respectively) than in immunocompetent women (1.6% and 0.8%, respectively) (*p* = 0.001).

**Conclusions:**

These results may reflect the increased risk of HPV persistent infection and disease progression in SOT women due to chronic immunosuppression.

## Introduction

Cervical cancer (CC) is the fourth most frequent cancer in women in the world, with estimated 570,000 new cases each year and 311,000 deaths in 2018 [1]. In Brazil, CC is the third most frequent cancer among women, with estimated 16,710 new cases per year and estimated incidence rate of 15.38 per 100,000 women [2]. In 2018, 6,526 deaths due to CC were registered, with mortality rate of 6.1 per 100,000 women [2]. Due to the lack of access to adequate CC screening, most Brazilian patients (60.1%) present with locally advanced or advanced CC at diagnosis [3]. Treatment of CC is also delayed, with most patients (58%) receiving their first treatment more than 60 days after diagnosis; early deaths (at the end of the first line treatment) are common (11%) [3].

Virtually all CC and most high-grade cervical cancer precursor lesions are associated with persistent infection with high-risk (HR) oncogenic Human Papillomavirus (HPV). HPV types 16, 18, 33, 45, 52 and 58 are responsible for about 90% of CC [1]. Low-risk HPV are rarely associated to CC, in general only among immunosuppressed women.

Solid organ transplant (SOT) recipients are at increased risk of persistent HPV infection and disease [4]. Several studies have reported high risk of CC in transplanted women, with standardized incidence ratio (SIR) of 30.7 (95% confidence interval [CI], 6.3–89.8) among liver transplanted women [4]; 25.3 (95%CI, 9.3–55.0) in kidney transplanted women [5] and 14.3 (95% CI, 1.7–51.5) in heart transplanted women [6]. SOT recipients also have higher risk of other HPV-related anogenital precursor lesions and cancers. A Danish cohort based on a nationwide nephrology register (from 1990 to 2015) found kidney transplant recipients (KTR) had increased hazard ratio (HR) of cervical (HR = 2.1, 95%CI 1.7–28), vaginal (HR = 35.0, 95%CI, 13.9–87.7), vulvar (HR = 16.4, 95%CI 10.4–25.8), penile (HR = 21.9, 95%CI 11.1–43.8) and anal (women: HR = 51.1, 95%CI 28.0–93.0 and men: HR = 39.0, 95%CI 16.7–91.1) intraepithelial neoplasia grade 2/3 (IN2/3) [7]. Progression of precursor lesions to cancer may be faster, as found in a follow-up study of KTR diagnosed with HPV-related lesions in the anogenital area [8].

Brazil ranks second among 35 countries in absolute numbers of kidney and liver transplantations, with about 95% of SOT financed by the public health system (*Sistema Único de Saúde*, *SUS*) [9]. SOT annual numbers reached 6,283 (30.1 per million population) kidney transplantations, 2,245 (10.8 pmp) liver, 173 (0.8 pmp) pancreas, 380 (1.8 pmp) heart and 106 (0.5 pmp) lung transplantations, in 2019 [9]. The Brazilian Guidelines for Cervical Cancer Screening recommends more frequent cytopathological examination of immunosuppressed women, with semiannual intervals in the first year and, if normal, annual follow-up as long as the immunosuppression is maintained [10]. Quadrivalent HPV (4vHPV) vaccine is recommended and freely available at SUS for all immunosuppressed men aged 9 to 26 years and for immunosuppressed women aged up to 45 years. However, data on CC and precursor lesions among SOT recipients are scarce in Brazil.

We conducted a prospective study to determine the safety and immunogenicity of HPV vaccination among adult female SOT recipients, compared to immunocompetent women of the same age in Sao Paulo, Brazil. In this paper, we present the cross-sectional analysis of HPV-related findings from study participants at enrolment in the cohort. This cross-sectional study aimed to assess the fraction of potentially vaccine-preventable disease by determining HPV seroprevalence, prevalence of cervical HPV-DNA, and HPV-related cytological lesions among SOT recipients compared to immunocompetent women of the same age.

## Methods

### Study populations and specimen collection

This cross-sectional study was conducted at the Reference Center for Special Immunobiologicals (*Centro de Referência para Imunobiológicos Especiais*, *CRIE*) of the Hospital das Clinicas da Faculdade de Medicina da Universidade de Sao Paulo (HC-FMUSP), from July 12, 2017 to October 16, 2018.

We enrolled SOT and immunocompetent women aged 18 to 45 years at the Kidney, Liver, Heart and Lung Transplantation Outpatient Clinics, and CRIE of HC-FMUSP, for a study that evaluated the immunogenicity and safety of quadrivalent HPV (4vHPV) vaccine in SOT women in comparison to immunocompetent women. Inclusion criteria for SOT women were: having had a transplantation at least six months earlier and being on immunosuppressive treatment. For immunocompetent women, the inclusion criteria were: absence of immunocompromise due to drugs or disease. Exclusion criteria for both groups were: pregnancy or breast-feeding; any other causes of immunocompromise, such as HIV/AIDS, cancer or primary immunodeficiency; biological therapy; heart, lung, liver, kidney or neurologic severe disease; history of genital warts or any HPV-related cervical, vaginal or vulvar lesions, as reported by the candidates; and previous HPV vaccination. Rapid HIV and pregnancy tests were performed for all candidates at enrollment.

The sample size calculation was based on the comparison of HPV seroconversion rates for each 4vHPV vaccine type in SOT and immunocompetent women. Based on the design and results of a previous comparative HPV vaccination study conducted among SOT recipients and non-immunosuppressed women in Canada [11], a sample size of 109 women in each group was sufficient to detect clinically significant differences on post-vaccination seroconversion rates (10%) between the two groups, with a power of 80% and precision of 5%, assuming 10% of losses in post-vaccination follow-up.

The following data were collected: age, color, educational level, age at menarche, age at sexual debut, sexual orientation, number of sexual partners, number of pregnancies, history of contraception and previous sexually transmitted infections (STIs), known comorbidities and, for SOT women, immunosuppressive drugs and regimens used. Body mass index (BMI) was measured for all women. A 5mL venous blood sample was collected for HPV serology. For women reporting having initiated sex, a speculum examination was performed by a trained nurse or physician during which, a cytobrush was used to sample the ecto/endocervical junction and inserted into a liquid-based cytology (LBC) vial (BD SurePath^™^, TriPath, Burlington, NC, USA) and transported daily, at room temperature to the laboratory.

### Laboratory testing

LBC samples were vortexed for 20 seconds and processed by the fully automated BD Totalys^®^ system, composed of a multiprocessor that integrates the pre-processing of the cytology sample with the molecular test. It uses 9 mL of SurePath^®^ liquid medium and performs "cell enrichment", that is, the removal of possible interferences and the formation of epithelial cells pellet in the SlidePrep^™^ equipment. Following, the pre-processed material for oncotic cytology was sent to the BD Totalys SlidePrep^™^ automated medium, for preparing and staining the slide. Cytology reading was performed at Fundacao Oncocentro de Sao Paulo (FOSP) laboratory, a public reference center for cytopathology. All cases were analyzed by cytotechnicians. In case of suspected abnormalities, they were reviewed by experienced cytologists and anatomopathologists. The results were reported according to the Bethesda System for reporting cervical cytology: negative for intraepithelial lesion or malignancy; atypical squamous cells of undetermined significance (ASC-US); atypical squamous cells that cannot exclude high-grade squamous intraepithelial lesion (ASC-H); low-grade squamous intraepithelial lesion (LSIL) (encompassing: HPV/mild dysplasia/CIN 1) and high-grade squamous intraepithelial lesion (HSIL) (encompassing: moderate and severe dysplasia, CIS; CIN 2 and CIS 3); atypical glandular cells favor neoplastic and endocervical adenocarcinoma in situ [12]. All participants with precursors lesions detected in this study were referred to a gynecologist to be treated.

Cervical samples were screened for HPV-DNA using PapilloCheck^®^ (Greiner Bio-One, UK), a polymerase chain reaction (PCR)-based DNA microarray system that can individually identify 25 HPV types, of which 12 high-risk (HR) HPV genotypes (16, 18, 31, 33, 35, 39, 45, 51, 52, 56, 58, 59) and 13 low-risk HPV types (6, 11, 40, 41, 42, 43, 44/55, 53, 66, 68, 70, 73 and 82). The test cannot distinguish between HPV 55 and 44 due to cross-reactivity. The test was performed according to the manufacturer’s instructions at the Laboratory of Medical Investigation / Virology (LIM-52), FMUSP.

Type-specific anti-HPV antibodies were detected using a multiplexed heparin-pseudovirion serology assay (PsV-Luminex). In this test, pseudovirions containing both L1 and L2 capsid proteins of eight HPV types (6, 11, 16, 18, 31, 33, 52 and 58) were used to detect type-specific anti-HPV antibodies, as previously described [13]. The test was performed at the Department of Laboratory Medicine, Karolinska Institutet, Sweden. The whole blood was stored at room temperature for no more than 8 hours. After centrifugation, the serum samples were aliquoted, then stored and transported at -20°C and analyzed all together at once. To define seropositivity for this study population, cut-off was calculated independently for each HPV type by analyzing the mean fluorescence intensity unit (MFI) values for 100 serum samples of Brazilian children aged 2 to 10 years. The global HPV LabNet recommended cut-off algorithm was applied: mean MFI value of a negative control serum panel plus 3 standard deviations. If the calculated cut-off value was less than 400 MFI, the 400 MFI was used as cut-off. Samples from SOT recipients were randomized and tested next to the samples from immunocompetent women.

### Statistical analysis

The data bank was built at REDCap 9.8.5 **- ©** 2020 (Vanderbilt University, Nashville, USA) and the statistical analyzes was conducted using R for Windows, version 3.6.1. Chi-squared test or Fisher’s exact test was used, as appropriate, for comparisons of categorical variables (HPV seroprevalence and prevalence and cervical intraepithelial lesions prevalence) between the two groups. Logistic regression analysis was used to analyze risk factors for HPV in both groups. Associations are expressed as odds ratio (OR) and their 95%CI. Statistical significance was set at p<0.05.

The study was approved by the Ethics Committee for Analysis of Research Projects (CAPPesq) of the HC-FMUSP (CAAE 66795817.9.0000.0068). All participants provided written informed consent.

## Results

We enrolled 125 SOT women (68 kidney, 4 kidney and pancreas, 28 liver transplanted, 17 lung and 8 heart transplanted) and 132 immunocompetent women aged 18 to 45 years. Table 1 shows the participants’ demographic and clinical characteristics. SOT women were slightly older than immunocompetent participants (mean age, 35.0 and 32.5 years, respectively); and this difference was statistically significant (p = 0.002). The immunocompetent women had higher educational level as compared to the SOT group (mean 14.3 years and 10.8 years of schooling, respectively, p<0.001). Use of hormonal contraceptive, number of lifetime partners, number of pregnancies and smoking were similar in both groups. Comorbidities, such as arterial hypertension, diabetes and dyslipidemia, were more frequent in the SOT group (69.6%) as compared to immunocompetent women (33.3%) (p<0.001). Most participants (96.9%) did not report any previous sexually transmitted infection (STI) diagnosis.

**Table 1:** Demographic and clinical characteristics of the study participants.

	Solid organ transplanted women (N = 125)	Immunocompetent women (N = 132)	*p-value*
**Age (years)**			**0.002**
Mean (SD)	35.0 (6.8)	32.5 (6.3)	
Median (minimum—maximum)	36.97 (18.53–45.33)	32.95 (18.15–45.58)	
**Color, n (%)**			0.394
White	64 (51.2)	74 (56.1)	
Black	58 (46.4)	52 (39.4)	
Asian	3 (2.4)	6 (4.5)	
**Years of schooling**			**<0.001**
Mean (SD)	10.8 (3.6)	14.3 (3.0)	
Median (minimum—maximum)	11.00 (0–17)	15.00 (3–22)	
**Age at menarche (years)**			0.306
Mean (SD)	12.7 (1.9)	12.5 (1.6)	
Median (minimum—maximum)	13 (8–18)	12 (9–18)	
**Age at sexual debut (years)**			0.443
Mean (SD)	18.2 (3.8)	18.5 (3.1)	
Median (minimum—maximum)	18 (12–33)	18 (12–30)	
**Number of lifetime sexual partners (%)**			
0	13 (10.4)	5 (3.8)	0.949
1–2	51 (40.8)	43 (32.6)	
3–5	46 (36.8)	59 (44.7)	
≥6	15 (13.0)	24 (18.1)	
Unknown	0	1 (0.8)	
**Current sexual partnership, n (%)**			
Heterosexual	93 (75.0)	100 (76.3)	0.364
WSW*	1 (0.8)	4 (3.1)	
None	30 (24.2)	27 (20.6)	
**Number of Pregnancies (%)**			0.358
0	67 (54.9)	79 (60.3)	
1	26 (21.3)	30 (22.9)	
2	13 (10.7)	14 (10.7)	
≥3	16 (13.1)	8 (6.2)	
**Current Contraceptive use, n (%)**			
None	91 (72.8)	72 (54.5)	**0.002** *
Hormonal	30 (24.0)	51 (38.6)	
Intrauterine device (IUD)	3 (2.4)	0	
Hormonal and IUD	1 (0.8)	9 (6.8)	
**History of sexually transmitted infections, n (%)**			
None	121 (96.0)	128 (97.0)	0.697*
Hepatitis B	1 (0.8)	0	
Genital herpes	1 (0.8)	2 (1.5)	
Unknown	2 (1.6)	2 (1.5)	
**Smoking (%)**			0.181
Never smoked	115 (92.0)	116 (87.9)	
Current smoker	2 (1.6)	8 (6.1)	
Ex-smoker	8 (6.4)	8 (6.1)	
**Comorbidities, n (%)**			**<0.001**
Arterial hypertension	49 (39.2)	5 (3.8)	
Diabetes	17 (13.6)	1 (0.8)	
Hypothyroidism	8 (6.4)	5 (3.8)	
Dyslipidemia	10 (8.0)	1 (0.8)	
Other	41 (32.8)	47 (35.6)	
None	38 (30.4)	88 (66.7)	
**Body mass index (kg/m** ^**2**^ **)**			0.526
Mean (SD)	25.7 (4.8)	25.3 (4.8)	
Median (minimum—maximum)	25.64 (15.06–39.21)	25.02 (17.22–39.91)	
**Type of solid organ transplant, n (%)**			
Kidney	68 (54.4)	-	
Kidney and pancreas	4 (3.2)		
Liver	28 (22.4)		
Lung	17 (13.6)		
Heart	8 (6.4)		
**Time since transplantation** (years)			
Mean (SD)	5.55 (4.98)		
Median (minimum—maximum)	4.02 (0.52–27.72)		

* Comparison between groups done based on categorically grouped Yes / No responses.

SD, standard deviation; WSW, women who have sex with women.

For SOT participants, the mean time since transplantation was 5.55 years. The conditions that led to organ failure and transplantation are described in the S1 Table. The immunosuppressive drugs used by the participants at the enrolment (S2 Table) varied according to the organ transplanted. The most frequent immunosuppressive drugs were Tacrolimus (88%), corticosteroids (84%) and Mycophenolate mofetil (MMF, 66.4%); and the most frequent immunosuppressive regimen were MMF plus Tacrolimus and Prednisone (64/125 or 51.2%), followed by Azathioprine plus Tacrolimus and Prednisone (14/125 or 11.2%).

Cervical samples for HPV-PCR and liquid-based cytology were collected from 113 SOT and 127 immunocompetent women who had initiated sexual activity. Results of HPV PCR and HPV serology are shown in Table 2. HPV-DNA was more frequently detected in SOT (29.6%) than in immunocompetent women (20.2%), but this difference was not statistically significant (p = 0.112).Current infection with one or more HR-HPV was significantly more frequent in SOT (19.4%) than in immunocompetent women (7.9%) (p = 0.014). Simultaneous infection with two or more HR-HPV was found in 3.1% of SOT and 0.9% of immunocompetent women (p = 0.434). The frequency of low-risk HPV was similar in SOT and immunocompetent women (10.2% and 8.8%, respectively, p = 0.772). The frequency of HPV types detected are shown in S3 Table. HPV types more frequently detected were 51 (in 6 women of each group), 53 (in 3 SOT and in 4 immunocompetent women), 16 (4 SOT and 2 immunocompetent women) and 68 (4 SOT and 1 immunocompetent women). HPV types included in 4vHPV vaccine (6, 11, 16 and 18) were detected in 8 SOT and 2 immunocompetent women, whereas HPV types included in the 9vHPV vaccine (6, 11, 16, 18, 31, 33, 45, 52 and 58) were detected in 12 SOT and 5 immunocompetent women.

**Table 2:** Results of HPV-DNA PCR in cervical samples and HPV serology (PseudoVirion-Luminex), according to study population.

	SOT women, n (%)	Immunocompetent women, n (%)	*P-value*
**Cervical HPV DNA** ^1^	**N = 99**	**N = 114**	
Negative	70 (70.7)	91 (79.8)	0.122
Positive	29 (29.6)	23 (20.2)	
≥1 HR* genotype	19 (19.4)	9 (7.9)	**0.014**
Multiple HR genotypes	3 (3.1)	1 (0.9)	0.434
≥ 1 LR** genotypes	10 (10.2)	10 (8.8)	0.772
Multiple HR or LR types	8 (8.1)	3 (2.6)	
Unknown HR/LR^#^	0	4 (3.5)	
**HPV serology** ^##^ ^**+**^	**N = 105**	**N = 119**	
Negative	38 (36.2)	48 (40.3)	0.524
Positive	67 (63.8)	71 (59.7)	
≥1 HR genotype	59 (56.2)	64 (53.8)	0.718
Multiple HR genotypes	25 (23.8)	31 (26.1)	0.699
≥1 LR genotypes (6, 11)	30 (28.6)	33 (27.7)	0.889
2vHPV vaccine types^&^	34 (32.4)	43 (36.1)	0.555
4vHPV vaccine types^&^	49 (46.7)	59 (49.6)	0.663
9vHPV vaccine types^&^	67 (63.8)	71 (59.7)	0.925

^1^ Missing results for 15 (13.3%) SOT and 13 (10.5%) immunocompetent women.

*HR genotypes tested by PCR: alpha9 types (16, 31, 33, 35, 52, 58, 68), alpha7 types (18, 39, 45, 56, 59).

**LR genotypes tested by PCR: 6, 11, 40, 41, 42, 43, 44/55, 53, 66, 68, 70, 73 and 82.

^#^ The test does not differentiate these HPV types.

^**##**^HR genotypes tested by serology: alpha9 types (16, 31, 33, 52, 58), alpha7 types (18, 45).

^&^Positive serology for one or more vaccine types.

HPV serology was performed for 105 SOT and 119 immunocompetent women. Seropositivity for at least one HPV type was observed in most women: 63.8% of SOT and 69.7% of immunocompetent women (Table 2). This difference was not statistically significant (p = 0.524). Seropositivity for at least one HR-HPV was similar in both groups (56.2% vs. 53.8%, p = 0.718). There were no statistically significant differences between the two groups regarding seropositivity for multiple HR genotypes, low-risk HPV or vaccine types (Table 2). Table 3 shows seroprevalence by HPV type in SOT and immunocompetent women. In addition to HPV 6, 11, 16 and 18, we found HPV 52 and 58 were quite frequent in both groups.

**Table 3:** HPV seroprevalence distribution, according to HPV genotype and study population.

HPV type	Solid organ transplanted women (N = 105) n (%)	Immunocompetent women (N = 119) n (%)
**6**	19 (18.1)	28 (23.5)
**11**	27 (25.7)	11 (9.2)
**16**	28 (26.7)	32 (26.9)
**18**	16 (15.2)	24 (20.2)
**31**	9 (8.6)	13 (10.9)
**33**	11 (10.5)	12 (10.1)
**52**	24 (22.9)	23 (19.3)
**58**	17 (16.2)	15 (12.6)
**Negative**	38 (36.2)	48 (40.3)

Table 4 shows the results of liquid-based cytology. SOT participants had higher prevalence of HPV-related cervical lesions (LSIL and HSIL) compared to immunocompetent women (15% versus 2.4%, p = 0.001).

**Table 4:** Results of liquid-based cytology, by study population.

Cytology*	Solid organ transplanted women (N = 113) n (%)	Immunocompetent women (N = 127) n (%)	*P-value*
Normal cytology / Benign cellular changes	82 (72.6)	107 (85.6)	
ASC-US	11 (9.7)	7 (5.6)	
ASC-H	1 (0.9)	8 (6.4)	
LSIL	11 (9.7)	2 (1.6)	0.001*
HSIL	6 (5.3)	1 (0.8)	
Unknown	2 (1.8)	2 (1.6)	

Note: Cervical samples collected only for women who had initiated sexual activity.

*ASC-US = atypical squamous cells of undetermined significance; ASC-H = atypical squamous cells, cannot exclude high-grade squamous intraepithelial lesion; LSIL = low-grade squamous intraepithelial lesion; HSIL = high-grade squamous intraepithelial lesion.

*Statistical analysis compared LSIL+HSIL vs. Normal cytology / Benign cellular changes.

Multivariate logistic regression analysis of risk factors for cervical HPV with a variable identifying SOT and controls was performed. Only variables with p<0.2 in the bivariate analyses (S4 Table) were included in the multiple model: group (SOT or immunocompetent), years of schooling, smoking, hypothyroidism, dyslipidemia and body mass index. At first, we included all variables in the model and then took them out one by one, beginning with those with high p-value until the final model, which showed significant association of HPV detection with lower educational level (OR 0.9, 95%CI 0.82–0.98, *p* = 0.013) and lower body mass index (OR 0.9, 95%CI 0.83–0.97, *p* = 0.005). SOT was not associated to a higher risk of HPV-DNA positivity in cervical samples in this study (*p* = 0.993).

In SOT women, there were no statistically significant differences in cervical HPV detection according to time since transplantation (comparing <5 years and ≥5 years since transplantation, p = 0.769), age at transplantation (comparing women aged <18 years and ≥18 years at transplantation, p = 0.499), type of organ transplanted (comparing liver transplant recipients with all other, p = 0.15) and immunosuppressive regimens (comparing MMF + tacrolimus + corticoids with all other immunosuppressive regimens, p = 0.621) (S5 Table).

## Discussion

This study compared SOT recipients and immunocompetent women aged 18 to 45 years, who denied previous HPV infection and related-lesions at the enrolment. HPV screening by PCR found a greater prevalence of HPV infection in SOT (29.6%) than in immunocompetent women (20.2%), but this difference was not statistically significant. But HR-HPV prevalence was significantly higher in SOT (19.4% vs 7.9%, p = 0.014). In addition, HPV serology demonstrated that most women (63.8% SOT and 59.7% immunocompetent) were seropositive for at least one HPV type, suggesting previous HPV exposure, despite absence of previous HPV related infection/diseases as reported by the participants. We also found a 5-fold greater prevalence of LSIL and HSIL in SOT compared to immunocompetent women of the same age range (15% vs. 2.4%). These results suggest similar exposure / HPV infection in both group, with higher HPV persistent infection in SOT women, resulting in higher frequency of HPV-related precursor lesions.

A systematic review (SR) with meta-analysis of the HPV prevalence in Brazil that excluded studies in people living with HIV/AIDS, found an overall cervical HPV prevalence of 25.4% (95%CI, 22.7–28.3%) [14], comparable to our results (24.5%). However, in our study, when separated by participants’ group, HPV prevalence was somewhat higher in SOT recipients (29.6%). HR-HPV prevalence was higher in the SR than our finding in immunocompetent women (17.6% and 7.9%, respectively). Prevalence of HPV16 and 18 were lower in our study (overall, 2.8% for HPV16 and 0.5% for HPV18, considering the whole population) as compared to the SR (5.3% for HPV16 and 1.9% for HPV18). However, seropositivity to HPV16 (26.7% SOT and 26.9% immunocompetent women) and HPV18 (15.2% and 20.2%, respectively) was high in our study, demonstrating previous exposure to these genotypes. In our study, a great diversity of HPV types was found (S3 Table). HPV types 51, 53, 16 and 68 were the most frequent and coinfection with two or more HR or low-risk HPV types were detected in 8.1% of SOT recipients and 2.6% of immunocompetent women.

Another Brazilian study evaluated HR-HPV and cervical cytological abnormalities in 32 kidney transplanted women aged from 31 to 70 years [15], ie. an older population older than in this study. They found higher prevalence of ASC-H (3.1%) and LSIL (12.5%) than in our study (ASC-H 0.9% and LSIL 9.7%). However, HSIL was more frequent in our study (5.3% versus none) and they found one case of squamous cell carcinoma whereas we had none. Prevalence of HR-HPV was similar in both studies (22.7% in the kidney transplanted in the previous study and 20.4% in SOT recipients in our study). The previous study did not evaluate HPV seroprevalence and there was no immunocompetent comparison group [15].

Another study, in Denmark, evaluated cervical cytological abnormalities; HR-HPV prevalence, and HPV genotype distribution in kidney transplant recipients (n = 43), and found a lower prevalence of cytological changes (ASC-US and LSIL, 7%; HSIL and ASC-H, 4.7%) [16], compared to our findings (ASC-US and LSIL, 19.4%; HSIL and ASC-H, 6.2%). The Danish study found lower prevalence of HR-HPV (9.3%) in kidney transplanted women, as compared to 20.4% of SOT recipients in our study. It is not easy to compare results of studies conducted in such different countries and populations, but, in the Danish study, women were older (median, 54 years) than ours (median, 36.97 years) and CC screening coverage is high in Denmark (64.2% in women aged 23 to 65 years, in 2018 [17]. These may be some of the many differences between the two populations that may explain the higher prevalence of CC precursor lesions and greater HR-HPV prevalence in our study.

Another study, conducted in Poland [18] evaluated the prevalence of HR-HPV in 60 kidney transplanted women aged from 20 to 48 years (similar to our population), in comparison to healthy ones. Only women with a previous normal Pap exam within 12 months were included. Results diverge from ours: HR-HPV prevalence was higher in healthy women (25%) than in transplanted women (18.3%), whereas in our study these figures were 7.9% and 19.4%, respectively. As expected, having more than two sexual partners was a risk factor for HR-HPV.

We were not able to find any Brazilian study on HPV seroprevalence in SOT population. Three studies evaluated seroprevalence of HPV types included in 4vHPV vaccine in non-immunocompromised women in Brazil [19–21]. Two studies included a younger population (maximum 25 years old) which may explain the lower seropositivity (HPV16, 9% and 14.6%; HPV18, 7% and 15.2%) [19, 20], as compared to our findings (HPV16, 26.7% in SOT and 26.9% in immunocompetent; and HPV18, 15.2% in SOT and 20.2% in immunocompetent). The third study [21] had a similar age group to ours and similar seroprevalence of HPV16 (30.8%) and HPV18 (21.7%), but significant lower seropositivity for HPV6 (10.9% vs. 18.1% in SOT and 23.5% in immunocompetent women, in our study) and HPV11 (3.5% vs. 25.7% in SOT and 9.2% in immunocompetent women, in our study).

The strengths of our study include enrolment of different types of solid organ transplant recipients (kidney, kidney and pancreas, liver, lung and heart); one of the largest sample size than reported elsewhere in literature; the inclusion of an immunocompetent comparator group and HPV serology evaluation.

A limitation of this study is also its sample size, which was calculated to inform the primary aim of the parent cohort study, ie. to determine the immunogenicity of 4vHPV vaccine in SOT recipients. However, considering the shortage of data on HPV prevalence in SOT recipients, this study may provide additional relevant information. Another limitation was the inclusion of women who verbally declared that they had not had a previous genital HPV-related lesion, without presenting previous tests. Women may have voluntarily omitted information on their prior disease so as to benefit from free HPV vaccination offered by enrolling in this study, since the vaccine were not available in the Brazilian public health system (SUS) for most of them, elevating HPV seroprevalence rates found in our study. It was not possible to analyze subgroups of SOT recipients due to the small number of liver, lung and heart transplant.

Considering that immunosuppression increases the risk of persistent HPV infection and CC development, further studies on HPV prevalence in SOT recipients and other immunocompromised populations are needed. This study results demonstrate the need of public policies for screening HPV and HPV-related cervical lesions specific for this immunosuppressed population. In addition, HPV vaccination may be an essential preventive measure for immunosuppressed women.

## Supporting information

S1 Table Conditions that led to organ failure and transplantation, according to organ transplanted in 125 SOT recipients study participants. *NSAID = non-steroidal anti-inflammatory drug.
(DOCX)

S2 Table Immunosuppressive drugs and regimens used by the 125 solid transplanted women at enrolment. (DOCX)

S3 Table HPV types detected by PCR in cervical samples of solid organ transplant recipients and immunocompetent women included in the study. *Papillocheck test does not differentiate these HPV types.
(DOCX)

S4 Table Bivariate analysis of risk factors for cervical HPV detection. (DOCX)

S5 Table HPV detection in SOT women according to time since transplantation, age at transplantation, organ transplanted and immunosuppressive regimens. (DOCX)
