# Supplementary material for: Human Papillomavirus (HPV) seroprevalence, cervical HPV prevalence, genotype distribution and cytological lesions in solid organ transplant recipients and immunocompetent women in Sao Paulo, Brazil
Source: PLoS One. 2022 Jan 20;17(1):e0262724. doi: 10.1371/journal.pone.0262724 (PMC8775251; doi:10.1371/journal.pone.0262724)
Supplement: S1 Table — *NSAID = non-steroidal anti-inflammatory drug. (DOCX) [file pone.0262724.s001.docx]

**S1 Table**: Conditions that led to organ failure and transplantation, according to organ transplanted in 125 SOT recipients study participants

| **Type of transplant** | **Condition** | **N (%)** |
| --- | --- | --- |
| **Kidney (n=68)** | Hypertensive nephropathy | 16 (23.5) |
|  | Glomerulonephritis | 12 (17.6) |
|  | Idiopathic | 11 (16.2) |
|  | Lupus nephropathy | 9 (13.2) |
|  | Diabetes | 6 (8.8) |
|  | Polycystic disease | 2 (2.9) |
|  | Focal segmental glomerulosclerosis | 2 (2.9) |
|  | IgA nephropathy | 2 (2.9) |
|  | Complications of infections | 2 (2.9) |
|  | Renal calculus | 1 (1.5) |
|  | Cystinosis | 1 (1.5) |
|  | Diabetes / pyelonephritis / pre-eclampsia / NSAID | 1 (1.5) |
|  | Nephritis / renal calculus | 1 (1.5) |
|  | Single kidney | 1 (1.5) |
|  | Trauma | 1 (1.5) |
| **Kidney + pancreas (n=4)** | Diabetes | 4 |
| **Liver (n=28)** | Autoimmune hepatitis | 12 (42.9) |
|  | Acute fulminant hepatitis | 4 (14.3) |
|  | Chronic hepatitis C | 2 (7.1) |
|  | Biliary atresia | 2 (7.1) |
|  | Idiopathic cirrhosis | 2 (7.1) |
|  | Primary biliary cirrhosis | 1 (3.6) |
|  | Wilson’s disease | 1 (3.6) |
|  | Chronic hepatitis B | 1 (3.6) |
|  | Familial amyloid polyneuropathy | 1 (3.6) |
|  | Budd-Chiari syndrome | 1 (3.6) |
|  | Type III glycogenosis | 1 (3.6) |
| **Lung (n=17)** | Cystic fibrosis | 7 (41.2) |
|  | Bronchiectasis | 4 (23.5) |
|  | Lymphangioleiomyomatosis | 2 (11.8) |
|  | Bronchiolitis | 1 (5.9) |
|  | Primary ciliary dyskinesia | 1 (5.9) |
|  | Histiocytosis | 1 (5.9) |
|  | Pulmonary silicosis | 1 (5.9) |
| **Heart (n=8)** | Congestive heart failure due to Chagas’ disease | 4 |
|  | Pregnancy-related cardiomyopathy | 2 |
|  | Single ventricle | 1 |
|  | Hypertrophic cardiomyopathy / arrhythmia | 1 |

*NSAID = non-steroidal anti-inflammatory drug
