# Supplementary material for: Human Papillomavirus (HPV) seroprevalence, cervical HPV prevalence, genotype distribution and cytological lesions in solid organ transplant recipients and immunocompetent women in Sao Paulo, Brazil
Source: PLoS One. 2022 Jan 20;17(1):e0262724. doi: 10.1371/journal.pone.0262724 (PMC8775251; doi:10.1371/journal.pone.0262724)
Supplement: S2 Table — (DOCX) [file pone.0262724.s002.docx]

**S2 Table**: Immunosuppressive drugs and regimens used by the 125 solid transplanted women at enrolment.

| **Immunosuppressive drugs** | **Total (%)** |
| --- | --- |
| Tacrolimus | 110 (88.0) |
| Corticosteroids | 105 (84.0) |
| Mycophenolate mofetil (MMF) | 83 (66.4) |
| Azathioprine | 18 (14.4) |
| Cyclosporine | 13 (10.4) |
| Everolimus | 6 (4.8) |
| Sirolimus | 4 (3.2) |
| Chloroquine | 1 (0.8) |
| Leflunomide | 1 (0.8) |
| **Immunosuppressive regimen** | **Total (%)** |
| MMF + Corticoids + Tacrolimus | 66 (52.8) |
| Corticoids + Azathioprine + Tacrolimus | 14 (11.2) |
| Cyclosporine + MMF + Corticoids | 10 (8.0) |
| Tacrolimus | 9 (7.2) |
| MMF + Tacrolimus | 6 (4.8) |
| Corticoids + Tacrolimus | 4 (3.2) |
| Azathioprine + Tacrolimus | 3 (2.4) |
| Corticoids + Sirolimus + Tacrolimus | 3 (2.4) |
| Corticoids + Tacrolimus + Everolimus | 3 (2.4) |
| Cyclosporine + Corticoids | 1 (0.8) |
| Cyclosporine + Corticoids + Azathioprine | 1 (0.8) |
| MMF + Corticoids + Everolimus | 1 (0.8) |
| Corticoids + Everolimus + Leflunomide | 1 (0.8) |
| Cyclosporine | 1 (0.8) |
| Cyclosporine + Corticoids + Tacrolimus | 1 (0.8) |
| Tacrolimus + Everolimus | 1 (0.8) |
| Prednisone + Sirolimus + Tacrolimus + MMF | 1 (0.8) |
