# Supplementary material for: Human Papillomavirus (HPV) seroprevalence, cervical HPV prevalence, genotype distribution and cytological lesions in solid organ transplant recipients and immunocompetent women in Sao Paulo, Brazil
Source: PLoS One. 2022 Jan 20;17(1):e0262724. doi: 10.1371/journal.pone.0262724 (PMC8775251; doi:10.1371/journal.pone.0262724)
Supplement: S3 Table — *Papillocheck test does not differentiate these HPV types. (DOCX) [file pone.0262724.s003.docx]

**S3 Table**: HPV types detected by PCR in cervical samples of solid organ transplant recipients and immunocompetent women included in the study.

| **HPV types** | **Solid organ transplanted women with HPV-PCR+** | **Immunocompetent women with HPV-PCR+** |
| --- | --- | --- |
|  | **N= 29 (%)** | **N=23 (%)** |
| **51** | 4 (13.8) | 5 (21.7) |
| **16** | 4 (13.8) | 2 (8.7) |
| **68** | 3 (10.3) | 1 (4.3) |
| **39** | 1 (3.4) | 2 (8.7) |
| **53** | 0 | 3 (13.0) |
| **42** | 2 (6.9) | 0 |
| **44/55*** | 0 | 2 (8.7) |
| **56** | 1 (3.4) | 1 (4,3) |
| **18, 52, 53** | 1 (3.4) | 0 |
| **33** | 1 (3.4) | 0 |
| **33, 42, 51, 52** | 1 (3.4) | 0 |
| **11, 39, 42, 44/55*** | 1 (3.4) | 0 |
| **39/42** | 0 | 1 (4.3) |
| **45, 58** | 0 | 1 (4.3) |
| **31, 45** | 1 (3.4) | 0 |
| **40, 44/55*, 51** | 1 (3.4) | 0 |
| **52/53*** | 0 | 1 (4.3) |
| **52/56/59*** | 0 | 1 (4.3) |
| **53, 59** | 0 | 1 (4.3) |
| **35, 53, 66** | 1 (3.4) | 0 |
| **11, 44/55*56** | 1 (3.4) | 0 |
| **51, 56** | 0 | 1 (4.3) |
| **58** | 1 (3.4) | 0 |
| **59** | 1 (3.4) | 0 |
| **42/53/59** ***** | 0 | 1 (4.3) |
| **6** | 1 (3.4) | 0 |
| **53, 68** | 1 (3.4) | 0 |
| **70** | 1 (3.4) | 0 |
| **73** | 1 (3.4) | 0 |

*Papillocheck test does not differentiate these HPV types
