# Supplementary material for: Human Papillomavirus (HPV) seroprevalence, cervical HPV prevalence, genotype distribution and cytological lesions in solid organ transplant recipients and immunocompetent women in Sao Paulo, Brazil
Source: PLoS One. 2022 Jan 20;17(1):e0262724. doi: 10.1371/journal.pone.0262724 (PMC8775251; doi:10.1371/journal.pone.0262724)
Supplement: S4 Table — (DOCX) [file pone.0262724.s004.docx]

**S4 Table:** Bivariate analysis of risk factors for cervical HPV detection.

| Variable | *p* value |
| --- | --- |
| Age at baseline | 0.424 |
| Number of sexual partners | 0.570 |
| Group (SOT or immunocompetent) | 0.101 |
| Years of schooling | 0.016 |
| Smoking | 0.070 |
| Hypothyroidism | 0.117 |
| Dyslipidemia | 0.062 |
| Body mass index | 0.020 |
| Age at sexual debut (years) | 0.448 |
| Current contraceptive use | 0.254 |
