# Supplementary material for: Human Papillomavirus (HPV) seroprevalence, cervical HPV prevalence, genotype distribution and cytological lesions in solid organ transplant recipients and immunocompetent women in Sao Paulo, Brazil
Source: PLoS One. 2022 Jan 20;17(1):e0262724. doi: 10.1371/journal.pone.0262724 (PMC8775251; doi:10.1371/journal.pone.0262724)
Supplement: S5 Table — (DOCX) [file pone.0262724.s005.docx]

**S5 Table:** HPV detection in SOT women according to time since transplantation, age at transplantation, organ transplanted and immunosuppressive regimens.

|  | HPV-PCR | | |
| --- | --- | --- | --- |
|  | Negative | Positive | Not done |
| **Time since transplantation, in years* (n)** |  |  |  |
| <5 (68) | 40 | 17 | 11 |
| 5 – 9.99 (36) | 15 | 8 | 13 |
| ≥10 (16) | 12 | 2 | 2 |
| **Age at transplantation, in years* (n)** |  |  |  |
| <18 (12) | 5 | 1 | 6 |
| 18-29 (40) | 20 | 8 | 12 |
| 30-45 (68) | 42 | 18 | 8 |
| **Organ transplanted (n)** |  |  |  |
| Kidney (72) | 37 (67.3%) | 18 (32.7%) | 17 |
| Liver (28) | 19 (82.6%) | 4 (17.4%) | 5 |
| Lung (17) | 10 (66.7%) | 5 (33.3%) | 2 |
| Heart (8) | 4 (66.7%) | 2 (33.3%) | 2 |
| **Immunosuppressive regimen (n)** |  |  |  |
| MMF + Tacrolimus+ Corticoids (65) | 40 (61.5%) | 15 (23.1%) | 10 |
| Azathioprine + Tacrolimus + Corticoids (14) | 6 | 5 | 3 |
| MMF + Cyclosporine + Corticoids (10) | 3 | 4 | 3 |
| All others (36) | 21 (58.3%) | 5 (13.9%) | 10 |

* 5 participants without information on time since transplantation / age at transplantation.
